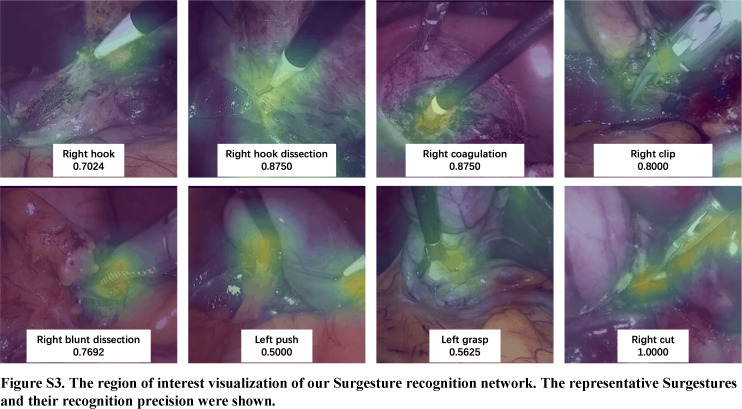# Decoding surgical skill: an objective and efficient algorithm for surgical skill classification based on surgical gesture features –experimental studies

**DOI:** 10.1097/JS9.0000000000000975

**Published:** 2023-12-11

**Authors:** Zixin Chen, Dewei Yang, Ang Li, Louzong Sun, Jifan Zhao, Jie Liu, Linxun Liu, Xiaobo Zhou, Yonghua Chen, Yunqiang Cai, Zhong Wu, Ke Cheng, He Cai, Ming Tang, Bing Peng, Xin Wang

**Affiliations:** a Department of General Surgery, Division of Pancreatic Surgery; b West China School of Medicine, West China Hospital of Sichuan University; c Chengdu Withai Innovations Technology Company, Chengdu; d Guang’an People’s Hospital, Guang’an; e Department of Hepatobiliary Surgery, Zigong First People’s Hospital, Zigong; f Chongqing University of Posts and Telecommunications, School of Advanced Manufacturing Engineering, Chongqing; g Department of General Surgery, Qinghai Provincial People’s Hospital, Xining, People’s Republic of China; h School of Biomedical Informatics, McGovern Medical School, University of Texas Health Science Center, Houston, USA

**Keywords:** laparoscopic cholecystectomy, machine learning, surgical gestures, surgical skill evaluation

## Abstract

**Background:**

Various surgical skills lead to differences in patient outcomes and identifying poorly skilled surgeons with constructive feedback contributes to surgical quality improvement. The aim of the study was to develop an algorithm for evaluating surgical skills in laparoscopic cholecystectomy based on the features of elementary functional surgical gestures (Surgestures).

**Materials and methods:**

Seventy-five laparoscopic cholecystectomy videos were collected from 33 surgeons in five hospitals. The phase of mobilization hepatocystic triangle and gallbladder dissection from the liver bed of each video were annotated with 14 Surgestures. The videos were grouped into competent and incompetent based on the quantiles of modified global operative assessment of laparoscopic skills (mGOALS). Surgeon-related information, clinical data, and intraoperative events were analyzed. Sixty-three Surgesture features were extracted to develop the surgical skill classification algorithm. The area under the receiver operating characteristic curve of the classification and the top features were evaluated.

**Results:**

Correlation analysis revealed that most perioperative factors had no significant correlation with mGOALS scores. The incompetent group has a higher probability of cholecystic vascular injury compared to the competent group (30.8 vs 6.1%, *P*=0.004). The competent group demonstrated fewer inefficient Surgestures, lower shift frequency, and a larger dissection-exposure ratio of Surgestures during the procedure. The area under the receiver operating characteristic curve of the classification algorithm achieved 0.866. Different Surgesture features contributed variably to overall performance and specific skill items.

**Conclusion:**

The computer algorithm accurately classified surgeons with different skill levels using objective Surgesture features, adding insight into designing automatic laparoscopic surgical skill assessment tools with technical feedback.

## Graphical abstract

**Figure:**
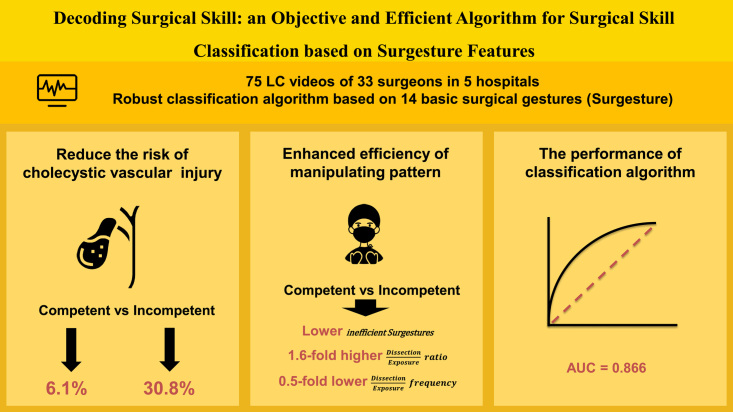

## Introduction

Highlights

- This study finds no correlation between surgeon-related information with their surgical skill.
- The incompetent group shows a higher risk of cholecystic vascular injury compared to the competent group.
- The competent group demonstrates a more efficient operative pattern of surgical gesture during the procedure.
- We develop a robust machine learning algorithm and achieve a high accuracy in binary surgical skill classification using surgical gesture features.
- Our results provide valuable insight for designing automatic surgical skill evaluation tools.

A large number of studies have revealed a strong correlation between a patient’s prognosis and a surgeon’s surgical expertise^1–3^. Greater surgical skill contributed to a lower complication rate, and each skill increment was associated with longer survival in oncological surgery^4^. Therefore, surgical skill determined the competency of surgeons and the quality of surgery. Nonetheless, despite receiving consistent training, practicing surgeons’ surgical performance varied substantially due to the long learning curve brought on by the intricacy of surgical practice^5^. To ensure a better quality of surgical treatment, an objective skill evaluation system is needed to screen poor-skilled practicing surgeons for continuing medical education and practice improvement.

Mounting institutes have conducted research on surgical skill assessment using surgical video^1,6^. Nowadays, numerous global structured rating systems, such as objective structured assessment of technical skills^7^ (OSATS) for open surgery, global operative assessment of laparoscopic skills^8^(GOALS) for laparoscopic surgery, and observational clinical human reliability assessment^9^ for identifying adverse events, are broadly utilized for surgical skill assessment. Although these rating scales provide reasonable feedback on surgeons’ skill, it still requires extensive review of the surgical video by many well-trained surgical specialists. These evaluation tools are time-consuming and susceptible to subjective bias, limiting their applicability and generalization. Consequently, an efficient, objective applicable surgical skill evaluation tool is urgently needed.

With the rapid development of artificial intelligence (AI), it has been increasingly applied in the field of surgery. Several algorithms demonstrate efficient and accurate identification of surgical phases, anatomy, and instruments^10–13^. Therefore, some research institutions are also investigating its feasibility in the field of surgical skill evaluation. Most studies have devoted their efforts to investigating kinematic information, such as surgical tools, hand, and eye motion tracking, to evaluate surgical skills^14,15^ or critical action^16^ to construct algorithms. Although these algorithms achieved reasonable results, they failed to provide surgeons with detailed feedback in terms of the specific ability for further improvement. In a previous study, we proposed the concept of Surgesture, which is defined as basic functional action (such as hook, grasp, and so on) performed by surgeons^17^, and discovered that Surgesture could not only be used to evaluate surgical skills but also provide feedback on surgeons’ technological shortcomings.

Therefore, there are two aims of our study: 1. To explore the factors related to surgical skill through analysis of demographic data and intraoperative data; 2. To construct an accurate, objective, and efficient algorithm to evaluate surgical skill based on Surgesture in the most wildly performed laparoscopic procedure—laparoscopic cholecystectomy (LC).

## Methods

### Datasets

The LC10000 data processing platform (http://lc10000-local.withai.com:10000, Figure S1, Supplemental Digital Content 1, http://links.lww.com/JS9/B547) was developed for the conservation, annotation, and analysis of LC videos across 18 hospitals. Surgeons from five hospitals in China participated in this study and submitted surgical videos and corresponding data to the platform from October 2020 to July 2021. To ensure consistency, we developed the following eligibility criteria. Inclusion criteria: (1) surgeons who have completed over 50 cases of LC and demonstrated proficiency by passing the learning curve^18^; (2) standard 3-port LC without any other concurrent process; (3) Parkland grading scale ≤2^19,20^. Exclusion criteria: (1) incomplete surgical video: low resolution, poor quality, or lack of critical surgical phase; (2) surgeon changed during the LC surgery. Detailed information about the surgeons and clinic data was collected, and the Committee of the Ethical Board reviewed and approved this study. Patients’ consent to video recording was obtained.

### Annotation

The 14 Surgestures and 7 LC surgical phases were defined according to our previous studies^17^. Mobilizing the hepatocystic triangle (MHT) and dissecting the gallbladder from the liver bed (DGB) were identified as the most critical and technique-challenge phases during LC^11^. We extracted the surgical videos corresponding to these two phases for the subsequent Surgestures annotation and algorithm construction. Two seniors and well-trained surgeons annotated the Surgestures by using our platform according to our previous definitions (Table S1, Supplemental Digital Content 2, http://links.lww.com/JS9/B548)^11^. The critical view of safety (CVS) scores, as well as abnormal events during the operation, were recorded. Any annotation disputes were resolved through consultation and discussion by a third senior surgeon.

### Rating and classification of surgical skill levels

Two senior surgeons reviewed all videos and rated them according to the modified GOALS (mGOALS) scoring system. The ‘Autonomy’ item was excluded from the rating because all surgeons had passed the learning curve and completed the procedure independently. The rating consistency was achieved by our training and intraclass correlation coefficient (ICC) testing (ICC >0.75 was regarded as a qualified rating). Skill levels were grouped into three classifications by mGOALS score of surgeons: top-level (scores within the 4th quantile); medium -level (scores between the 1st and 3rd quantile), and bottom-level (scores within the 1st quantile). Comparisons and correlation analysis of perioperative information and Surgesture data were conducted among the different skill level groups.

### Machine learning algorithm construction for skill level classification

In our previous study, Surgestures were found to be significantly correlated with surgical skill and could serve as objective indicators for skill level^17^. Given that, we developed a machine learning algorithm to distinguish the different levels of surgeons based on Surgesture data (Figure S2, Supplemental Digital Content 3, http://links.lww.com/JS9/B549). First, surgical videos were divided into the competent group (top and medium-level) and the incompetent group (bottom-level). Video data were augmented referring to the data augmentation approaches of the natural language process to equalize data and lead to robust and unbiased performance^21^. Surgesture features such as counts, duration, intervals (mean/max/min/SD), manipulation time (the duration of MHT and DGB), and shifting frequency of Surgestures were extracted and analyzed. The counts and durations of the ratio of dissection and exposure (D/E ratio), as well as the shift frequency of D/E Surgestures, were also included because the D/E ratio was proved to be a reliable indicator of surgical efficiency^22^ (Detailed in Table S2, Supplemental Digital Content 4, http://links.lww.com/JS9/B550). The selected features were used to develop this automatic surgical performance classification algorithm, which was validated using fivefold-crossing-validation and classic machine learning algorithms, such as Logistics Regression (LR), Support Vector Machine (SVM), Random Forest (RF), Gradient Boosted Decision Trees (GBDT), and Adaboost models. The performance was evaluated using the area under the receiver operating characteristic (ROC) curve (AUC). Feature importance mining was conducted to explore the weight coefficient of Surgestures in the surgical skills assessment. The algorithms were developed using scikit-learn 1.0.2, numpy 1.21.6, and pandas 1.3.5 in Python 3.7.

### Statistics

The data were presented by mean±SD or median (interquartile range). Kruskal–Wallis test and Student *t*-test were used to analyze the differences among skill classification groups. The χ^2^ test of independence was used to test the frequency distribution between groups. Kendall’s *W* test was used to test the consistency of the score, and Spearman correlation analysis was used to analyze the correlation between Surgestures and mGOALS scores. All statistical analyses were performed using SPSS 20.0 (IBM Corp).

## Results

### Basic information on surgeons, patients, and surgical videos

Our study involved a cohort of 33 surgeons from five hospitals, as presented in Table 1. Case experience varied significantly, ranging from 50 to over 2000, with 10 surgeons (30.3%) reporting experience ranging from 200 to 300 cases. Of the participating surgeons, 48.5% were attending, followed by the vice-chief (27.3%) and resident (21.2%). A total of 75 patients diagnosed with stone/cholecystitis/polyp underwent LC procedures. Through annotating and analysis of surgical video, 64 (85.3%) surgeries were classified as Parkland level 1, while 11(14.7%) were classified as Parkland level 2. The median time of the MHT and DGB phases was 11.18 (6.44) min. CVS was achieved in only 5(6.0%) patients, with a median score of 2 (1.5). The most common intraoperative event was liver thermal injury, which occurred in 68 (90.1%) cases, followed by cholecystic vascular injury (14.7%), and gallbladder rupture (6.7%). The median length of stay was 2 days, and no major complications after surgery.

**Table 1:** The demographic description of surgeons, videos, and patients.

Category	Statistics
Surgeon Info (33)	
Work years (Median, IQR)	8 (15)
Age (Means, SD)	38 (5.9)
Sex (Male/Female)	32/1
Hospital level (*N*, %)	
Ministerial level	8 (24.2%)
Prefectural and provincial	19 (57.6)
County level	6 (18.2)
Case experience (*N*, %)	
50=<x<100	4 (12.1%)
100=<x<200	3 (9.1%)
200=< x <300	10 (30.3%)
300=< x <400	3 (9.1%)
400=< x <500	3 (9.1%)
500=< x <1000	2 (6.1%)
1000=< x <2000	4 (12.1%)
>=2000	4 (12.1%)
Title (*N*, %)	
Resident	7 (21.2%)
Attending	16 (48.5%)
Vice-chief	9 (27.3%)
Chief	1 (3.0%)
Surgical videos (75)	
Operation time (Median, IQR)	11.18 (6.44)
Kinds of Surgestures (Median, IQR)	11 (2)
Parkland level	
PKL1	64 (85.3%)
PKL2	11 (14.7%)
CVS	
Total scores (Median, IQR)	2 (1.5)
Accomplishment (Total scores >4, %)	5 (6.0%)
Events	
Gallbladder broken	5 (6.7%)
Liver thermal injury	68 (90.1%)
Gastrointestinal injury	0
Bile duct injury	0
Cholecystic vascular injury	11 (14.7%)
Parietal peritoneum injury	0
Check for cystic stone	42 (56.0%)
Patient info (75)	
Sex (Male/Female)	13/62
Age (Mean, SD)	47.5 (13.8)
BMI (Mean, SD)	23.02 (2.75)
Weight (kg, Mean, SD)	58.4 (9.0)
Diagnose	
Gallbladder polyp	5 (6.7%)
Stone with/without chronic cholecystitis	47 (62.7%)
Stone with/without acute cholecystitis	22 (29.3%))
Acute cholecystitis	1 (1.3%)
ASA (I/II)	7/68
Drainage	1 (1.3%)
Length of stay (Median, IQR)	2 (2)
Complications	None

### mGOALS score skill classification

**Figure 1:**
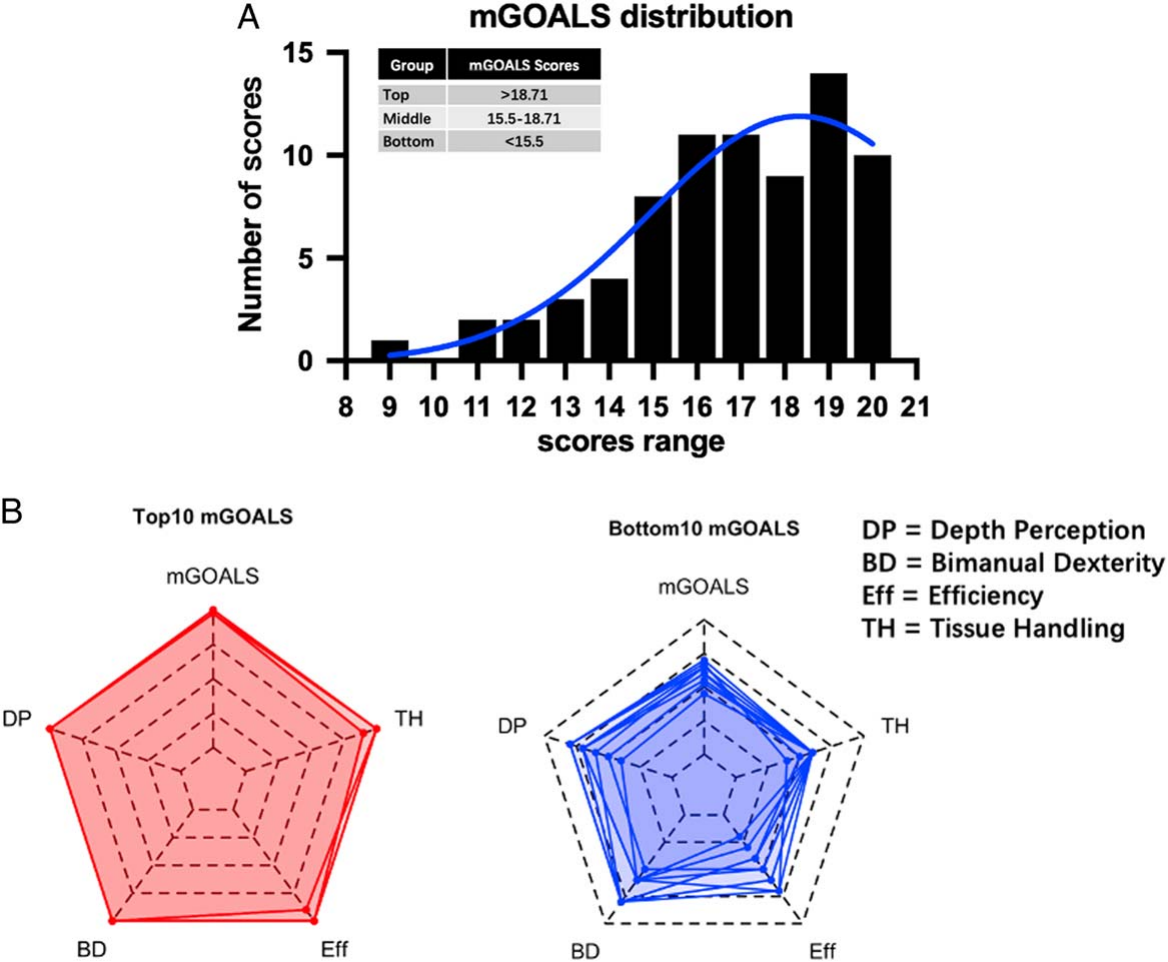
The mGOALS distribution of all videos and the representative top/bottom score composition. A. The distribution of mGOALS scores of all videos. Mean scores were used when one surgeon submitted several videos. The table in the left-up shows the cutoff scores (1st and 3rd quantile) among the groups. B. The left radar plot shows the top 10 video mGOALS scores composition while the right shows the bottom 10. BD, bimanual dexterity; DP, depth perception; Eff, efficiency; TH, tissue handling.

As shown in Figure 1A, mGOALS scores demonstrated a right-skewed distribution, which ranged from 9 to 20. The 1st and 3rd quantiles were 15.5 and 18.71, respectively. Accordingly, the participating surgeons were stratified into three groups according to mGOALS scores: top-level (scores greater than 18.71), medium-level (scores between 15.5 and 18.71), and bottom-level (scores lower than 15.5) (Fig. 1A). Radar plots further revealed that the top-level group scored ~5 in each skill item, while the bottom-level group’s scores varied from 1 to 4 in those items (Fig. 1B). Regarding surgical outcomes, the incompetent group had a significantly higher incidence of cholecystic vascular injury compared to the competent group (30.8 vs 6.1%) (Table 2). However, as surgical skills improved, the probability of intraoperative checks for cystic duct stones significantly decreased (65.4% vs 63.6% vs 25.0%) (Table S3, Supplemental Digital Content 5, http://links.lww.com/JS9/B551). Notably, our correlation analysis revealed that factors conventionally used to evaluate surgical skill, such as hospital level, case experience, age, title, and the achievement of CVS, did not demonstrate a significant correlation with the mGOALS score or individual item scores (Table S4, Supplemental Digital Content 7, http://links.lww.com/JS9/B552). The only exception was the number of work years, which displayed a significant positive correlation (R=0.373) with the depth perception scores (Table S4, Supplemental Digital Content 7, http://links.lww.com/JS9/B552).

**Table 2:** The comparison of cholecystic vascular injury and CVS achievement between the two group.

Group	Total	No	Yes	χ^2^	*P*
Cholecystic vascular injury					
Top and medium	49	46 (93.9%)	3 (6.1%)	8.245	0.004
Bottom	26	18 (69.2%)	8 (30.8%)		
CVS achievement					
Top and medium	49	42 (85.7%)	7 (14.3%)	0.699	0.403
Bottom	26	24 (92.3%)	8 (7.7%)		

### Surgesture features applied to construct a classification model

**Figure 2:**
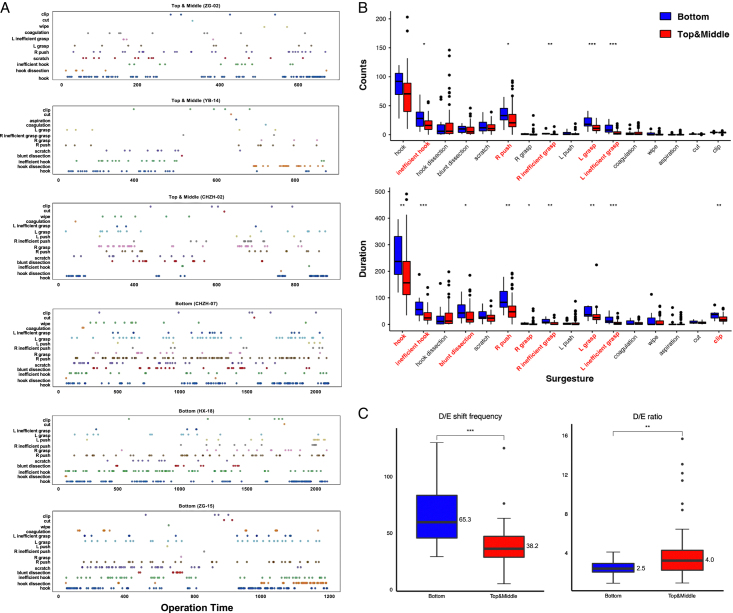
The analysis of Surgesture features between the two groups. A. The distribution of Surgesture as operation time. Three samples were randomly selected to demonstrate in the competent group (top three) and the incompetent group (bottom three), respectively. The dots represent the start time point of different Surgestures during MHT and DGB. B. The counts and duration comparison of all Surgestures between the two groups. C.The D/E shift frequency and D/E ratio comparison between the two groups. *, *P*<0.05; **, *P*<0.01; ***, *P*<0.001. Red, competent group; Blue, incompetent group.

In this study, we comprehensively analyzed Surgesture features with different skill levels, revealing significant differences between the competent and incompetent groups. As depicted in Figure 2A, the competent group demonstrated a more sparse and shorter distribution of Surgesture across the two critical phases during LC. Conversely, the incompetent group showed a higher count and duration of inefficient and exposure Surgestures, such as inefficient hook, inefficient grasp, push, and grasp (Fig. 2B). Moreover, the competent groups exhibited significantly shorter durations of hook and blunt dissection compared to the incompetent group. In addition, the D/E ratio of competent groups was 1.6-fold higher than that of the incompetent group (*P*<0.01), and the D/E frequency was approximately half that of the incompetent group. To develop an optimal surgical skill classification algorithm, we extracted a total of 63 multilevel features of all Surgestures, including counts (*n*=20), duration (*n*=20), intervals (*n*=12), shift frequency (*n*=3), and D/E classifications (*n*=7) of Surgestures and the manipulation time (1) (Detailed in Table S2, Supplemental Digital Content 4, http://links.lww.com/JS9/B550).

### The classification performance and further features mining

**Figure 3:**
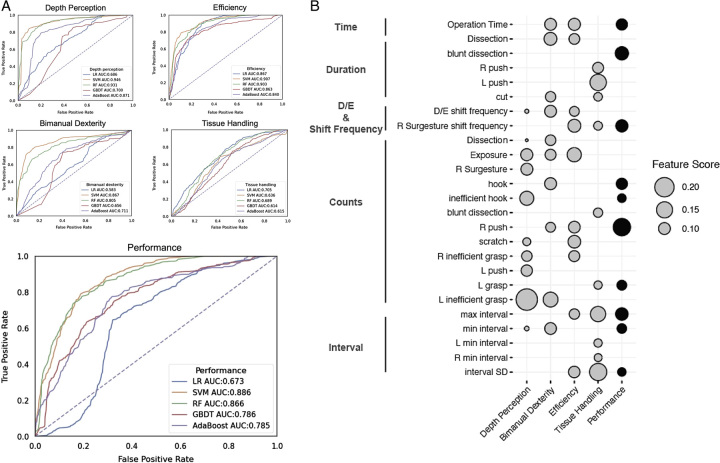
The prediction performance and the feature mining result. A. The AUC of different classification algorithms in the prediction of depth perception, bimanual dexterity, efficiency, tissue handling, and overall surgical skill, respectively. B. The top 10 features of classification models in each surgical skill item. *Y*-axis lists the features and its category. The circle size means the normalized feature scores.

The ROC curve in Figure 3 demonstrates the overall performance of the five models as well as four detailed skill items. The AUC of overall classification performance ranged from 0.673 to 0.886 across five models, among which SVM had the best performance (0.886). Besides, SVM also had the best performance in skill classification of depth perception (0.946), bimanual dexterity (0.867), and efficiency (0.907). However, tissue handling evaluation proved to be the most challenging item for algorithms, with the AUC ranging from 0.614 to 0.705 (Fig. 3A). To better understand the Surgesture features importance for skill classification, we calculated the top 10 features contributed to each classification model according to weight (Fig. 3B). In detail, the counts of left inefficient grasp were the most influential factor for depth perception (normalized feature score=0.2), while the classification of bimanual dexterity and efficiency particularly depended on manipulation time, duration of dissection, D/E shift frequency, and the counts of exposure Surgesture (Fig. 3B). Furthermore, the max/SD interval and shift frequency of right Surgestures have an important weight in the classification of efficiency (Fig. 3B). For tissue handling classification, both the duration of left push and max/SD interval had the most critical impact. Lastly, Surgesture features including the counts of right-hand push and left-hand grasp, manipulation time, duration of blunt dissection, shift frequency, and max/min/SD intervals had great contributions to overall performance classification (Fig. 3B).

## Discussion

Our study focused on LC, a common surgical procedure, and found significant differences in the skills of surgeons performing this routine operation. Notably, surgeon-related factors such as hospital levels, case experiences, title, and age did not appear to have an impact on overall surgical skill scores. However, we did observe a weak correlation between work years and the surgeon’s depth perception. To objectively evaluate surgical skill, we employed Surgesture, which revealed notable differences in performance among surgeons of varying skill levels. By utilizing 63 Surgesture features, we developed a robust assessment model for surgical skill, achieving an impressive overall performance with an AUC of 0.886. Furthermore, we observed that Surgesture features had varying effects on each rating item, providing valuable feedback on specific areas where surgeons can improve.

Numerous studies have demonstrated the vital role of surgical skills in determining surgical quality and patient outcomes. Brajcich *et al*. found that patients following colorectal tumor surgery by a highly surgical skilled surgeon probably enjoyed longer long-term survival^4^. In bariatric surgery, Birkmeyer *et al*.^2^ demonstrated that greater surgical skill was associated with reduced postoperative complications, reoperation rates, readmission rates, and emergency department visits. Our study further adds to this evidence by highlighting that even in routine and widely performed LC procedures, there is significant variation in surgical skills among surgeons. Besides, it is noteworthy that the incidence of cholecystic vascular injury was significantly lower incompetent surgeons than in incompetent surgeons, indicating that the surgeon’s surgical skill has a pervasive impact on patient prognosis that extends beyond the type or complexity of surgery. Consequently, identifying technical inadequacies and providing tailored feedback to enhance surgical skills is crucial for improving patient outcomes.

In order to achieve these goals, a variety of research teams have investigated numerous measures to quantify surgical skills, including surgeon-related factors and perioperative information. On the one hand, surgeon-related factors included age, work experience, case volume, hospital levels, etc. Among them, case experience is one of the most commonly used measures. Doneza *et al.* asked 108 obstetricians and gynecologists to perform tasks on a laparoscopic virtual reality simulator and showed a weak correlation among surgical volume, duration (r=−.032), and blood loss (r=−0.29). However, no difference in ovarian diathermy damage was observed regardless of case volume and fellowship-trained^23^. In addition, hospitals with larger surgical volumes had lower surgical mortality rates, many centers use annual surgical volume to assess whether a surgeon/hospital is qualified to perform a certain type of complex surgery^24,25^. Varban *et al.* ^26^ studied 25 surgeons with their 33 Laparoscopic Sleeve Gastrectomy videos and found no significant difference in mean age, years in surgery practice, or whether been practiced at a teaching hospital between high rating group and low rating group, but the high rating group have a larger case volume and shorter operative time. On the other hand, perioperative information including bleeding volume, operative duration, and postoperative complications has also been used to establish classification to enhance the quality of surgery^27^. However, these metrics are influenced by several factors (such as surgical difficulty and anatomical variation) and do not directly correlate with surgical skills, making it challenging to establish relevant classification criteria with surgical skills. In our study, we found that the skill rating, actually, has no significant correlation with their characteristics such as title, seniority, or case experience. This highlights the limitations in assessing surgical skills using surgeon-related factors or perioperative information.

The surgical field has recently witnessed the emergence of AI, which has the potential to revolutionize surgical training, assessment, and patient outcomes^28–30^. Video-based assessments using AI have shown promising results in surgical skill evaluation. Azari *et al*.^31^ used computer vision to capture kinematic data for measuring surgical skill in open surgery and the predicted results commensurate with the expert’s rating. Meanwhile, Lavanchy *et al*.^16^ developed a three-step machine learning method to identify and extract the localization of clip applier during LC and distinguish the good versus poor clip skill. They did achieve a relatively high accuracy of about 87%. Kitaguchi *et al*.^32^ divided 650 laparoscopic colorectal surgical videos into three groups by the means of skill rating scores±2SD, and they developed a 3D convolutional neural network model trained with about 1100 clips. These models achieved a mean accuracy of 75.0% in skill classification. However, these models have limitations in providing specific feedback or detailed information for the improvement of surgical skills, acting more like ‘black boxes^33^’ for skill evaluation.

The surgical actions are the specific mapping of surgical steps and tasks, and they are the direct embodiment of surgical skills. Recently, SAGES reached a consensus recommending four hierarchies of surgical video segmentation: phase, step, task, and action, emphasizing the critical role of surgical action in video data analysis and evaluation^34^. Since the Surgestures are the basic unit of all laparoscopic procedures with high accessibility and plasticity^17^, just like the ‘words’ in the language of surgery^35^, their utilization could be easily extended in other laparoscopic surgeries. In the present study, we identified a significant difference in the layers of Surgesture features among varied skilled surgeons. Nonetheless, the combination of AI and Surgestures holds promise in developing a surgical skill evaluation system with performance feedback immediately after an operation^36^.

In this pilot study, we initially stratified the Surgesture features into coarse and fine-grained according to their properties^17,22^. The dissection and exposure-related features were allocated to the coarse-grained category, while the specific Surgestures features were classified as fine-grained. Considering the complexity of the model structure, overfitting risk, topology fraction ability, interpretability, robustness, model bias, and other constraints, we process coarse-fine granularity data in layers with reference to the neural network. We employed classical machine learning models such as LR, SVM, AdaBoost, GBDT, and RF with different focuses on algorithm development and observed that the SVM-based SmartSkill evaluation system performed the best in the overall surgical skill assessment performance (AUC=0.886) and in the assessment of different skill dimensions. Moreover, the feature mining results reinforced the interpretability of Surgesture features used for classification. For instance, we found that left-hand grasp weighted the most in the depth precision rating, while right-hand frequency and exposure of Surgestures weighted most in the efficiency evaluation. With accumulated evidence highlighting the importance of preoperative training^37^, those findings might offer valuable insights into the orientation of surgical practice programs and reference for the surgeons tailoring their practice planning using simulators, like LapSim^38^. This will build the bridge to the hybridization of both preoperative training and postoperative assessment, holding a promising way of surgical skills enhancing.

### Limitation

Despite the promising potential of AI and Surgestures in evaluating surgical skills, there remain limitations that must be addressed. First of all, further external validation is necessary for evaluating the generalizability to a real-world setting of the model. We did not include complex LC surgery and other important factors related to surgical quality and safety (such as CVS achievement and check for cystic stones in our study). Complex surgery demands a combination of teamwork, skills proficiency, as well as experience in dealing with uncertainty (such as anatomical variations). Experienced surgeons are often expected to perform well in challenging LC surgeries. But it is well understood that high-quality surgery is the result of skills and decision-making transformed from experience, while in routine procedures like simple LC, it is easy to isolate the impact of surgical experience on skill evaluation. This might introduce bias in surgical skill evaluation, focusing on the standardization of basic surgical actions while overlooking the decision-making transformed from case experience. Furthermore, the manual annotation of Surgesture remains labor-intensive and time-consuming, our study also lacks a comprehensive definition of complex Surgestures, such as suturing and knot-tying, which poses a significant obstacle to enlarging its applicability to broader research on a national scale and various surgical procedures. To overcome these limitations, we are developing an automated recognition model of Surgesture based on computer vision technology and have verified its preliminary feasibility (Figure S3, Supplemental Digital Content 6, http://links.lww.com/JS9/B553). Meanwhile, we aim to integrate more comprehensive parameters in the follow-up work to further strengthen its performance and generalization.

## Conclusion

In summary, most of the currently available tools for assessing perioperative metrics and surgical skills still have various limitations such as high subjectivity, weak correlation, and poor feedback. There, we developed a machine learning model based on various Surgesture features and achieved an objective, accurate, and multidimensional assessment of skill in real surgery for the first time. Our findings provide valuable insight into designing a highly scalable assessment tool with strong technical feedback for all types of laparoscopic surgical skill assessment in the future.

## Ethical approval

The Ethics Committee on Biomedical Research, West China Hospital of Sichuan University approved this study (2020503).

## Consent

Written informed consent was obtained from the patient for publication of this case report and accompanying images. A copy of the written consent is available for review by the Editor-in-Chief of this journal on request.

## Sources of funding

This work was supported by the Fund of the High-Quality Development of Guang’an People’s Hospital (21FZ001), Key Project of Science & Technology Department of Sichuan Province (2022YFS0024), Regional Innovation Cooperation in Sichuan Province (2022YFQ0068).

## Author contribution

Z.C.: conceptualization, formal analysis, methodology, visualization, and writing – original draft; D.Y.: formal analysis, methodology, validation, and writing – original draft; A.L.: funding acquisition, investigation, project administration, resources, and supervision; L.S.: data curation, formal analysis, investigation, methodology, and validation; J.Z.: formal analysis, methodology, software, validation, and visualization; J.L.: data curation, funding acquisition, investigation, project administration, resources, software, and supervision; L.L.: data curation, formal analysis, investigation, and resources; X.Z.: conceptualization, investigation, project administration, resources, software, and validation; Y.C.: formal analysis, investigation, methodology, and resources; Y.C.: data curation, investigation, methodology, resources, supervision, and validation; Z.W.: data curation, formal analysis, investigation, methodology, supervision, and visualization; K.C.: funding acquisition, investigation, methodology, validation, and visualization; H.C.: data curation, formal analysis, investigation, and validation; M.T.: data curation, formal analysis, methodology, and validation; B.P.: conceptualization, funding acquisition, project administration, resources, supervision, and writing – review and editing; X.W.: conceptualization, funding acquisition, supervision, and writing – review and editing.

## Conflicts of interest disclosure

The authors declare that they have no financial conflicts of interest with regard to the content of this report.

## Research registration unique identifying number (UIN)

1. Name of the registry: not applicable.
2. Unique identifying number or registration ID: not applicable.
3. Hyperlink to your specific registration (must be publicly accessible and will be checked): not applicable.

## Guarantor

Xin Wang.

## Data availability statement

The video data of surgical gesture annotation are available upon request.

## Provenance and peer review

Not invited.

## Presentation

The abstract has been accepted as a quick shot presentation in the Scientific Forum at Clinical Congress 2023: October 22-25, 2023 in Boston, MA. Session: SF303 I Surgical Education V: Surgical Skills. The website for the conference is https://www.facs.org/for-medical-professionals/conferences-and-meetings/clinical-congress-2023/call-for-abstracts/.

## Supplementary Material

SUPPLEMENTARY MATERIAL

## Supplementary Material

**Figure:**
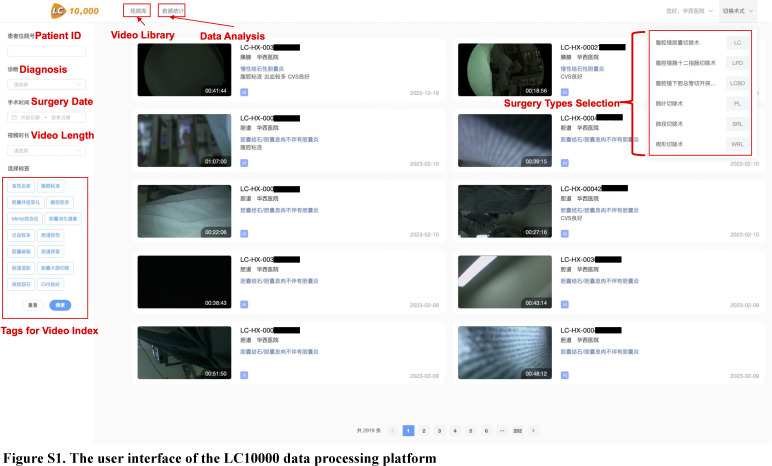


**Figure:**
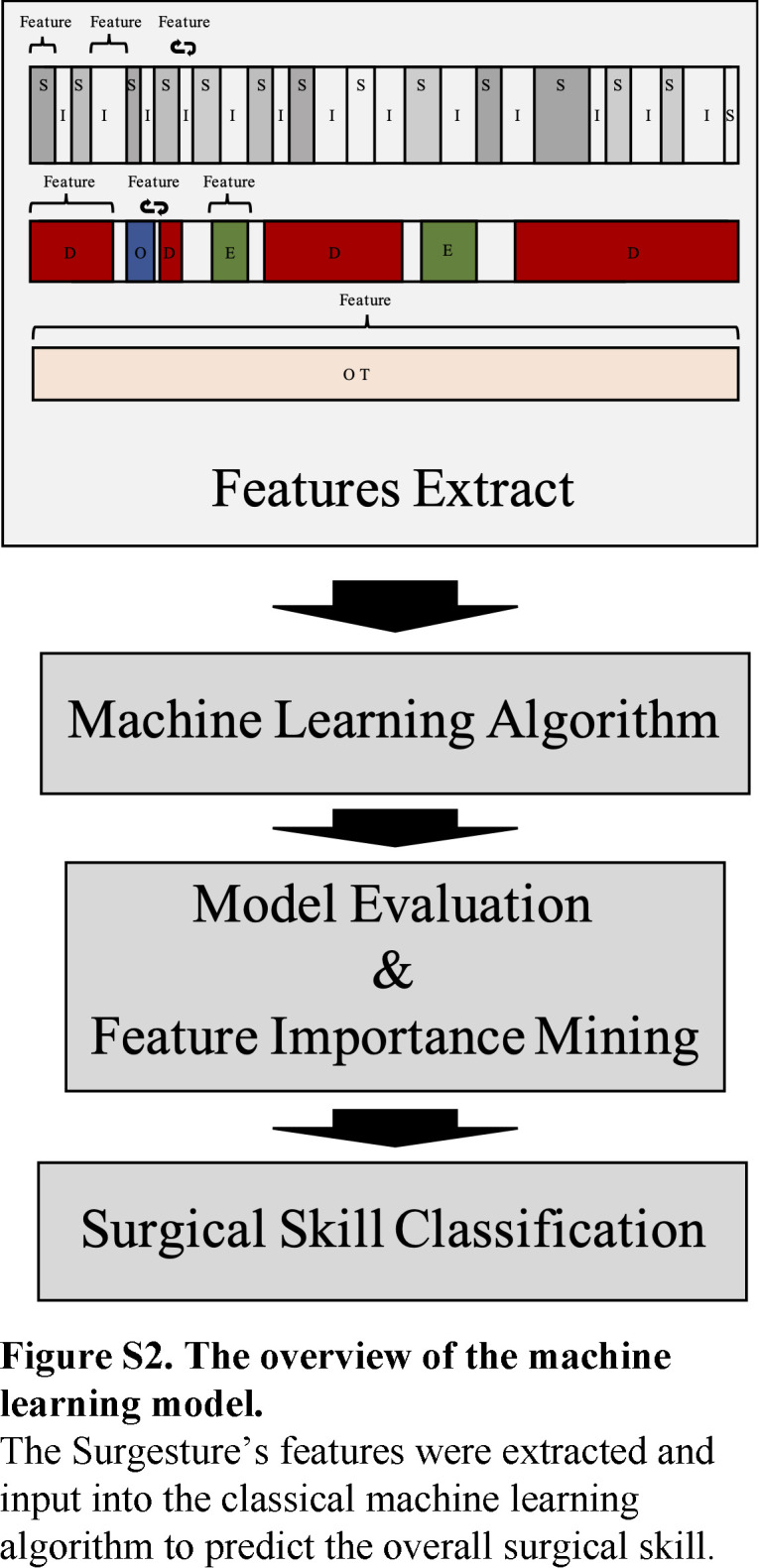


**Figure:**