# Supplementary material for: Decoding surgical skill: an objective and efficient algorithm for surgical skill classification based on surgical gesture features –experimental studies
Source: Int J Surg. 2023 Dec 11;110(3):1441–9. doi: 10.1097/JS9.0000000000000975 (PMC10942222; doi:10.1097/JS9.0000000000000975)
Supplement: SUPPLEMENTARY MATERIAL [file js9-110-1441-s002.docx]

**Table S1. The previous definition of 14 surgestures from our study^1^.**

| Surgestures | Definition | Start/End |
| --- | --- | --- |
| Hook | The tissue is hooked and is disconnected by the cautery hook | Start：the cautery hook begins to enter the anatomical plane  End：the cautery hook is activated and the tissue is disconnected |
| Hook dissection | The tissue is dissected by the back or tip of the cautery hook | Start：the back or tip of the cautery hook reaches the tissue  End：the cautery hook is activated and the tissue is dissected |
| Blunt dissection | The tissue is dissected and separated by forceps | Start：the instruments begin to enter the anatomical plane  End：the instruments exit the anatomical space |
| Inefficient hook | The tissue is hooked by cautery hook without disconnection or activation | Start：the cautery hook begins to enter the anatomical plane  End：the cautery hook leave/exit the tissue without activation or disconnection of the tissue |
| Grasp | The tissue/free item (e.g. clip, gauze) is grasped and displaced | Strat：the forceps reach the tissue/free items and grasp  End：the forceps loosen the tissue or out of sight (for 3s) |
| Push | For the purpose of exposure, the tissue is displaced by pushing or pressing | Start：the instruments reach the tissue/free items and displaced  End：the instruments leave the tissue/ free items or out of sight |
| Scratch | For the purpose of exposure, the tissue is scratched by instruments | Start：the instruments reach the tissue  End：end of the scratch action |
| Inefficient grasp | No operation was performed on repeated (more than twice) grasps or slips/misses within a short period of time | Start: the forceps reach the tissue/free items  End: the instruments leave the tissue/ free items |
| Coagulation： | For the purpose of hemostasis, the bleeding point is cauterized by electrocoagulation the cautery hook/coagulator | Start: the cautery hook/coagulator reach the tissue  End: the instruments finish the hemostasis and move to another place |
| Tamponade | For the purpose of compression hemostasis, the bleeding point is compressed by gauze or aspirator | Start: the gauze/aspirator reach the tissue  End: the gauze/aspirator leave the tissue |
| Aspiration | For the purpose of clean, the fluid/blood/bile or other substances is aspirated by aspirator | Start: the aspirator reaches the targets  End: the aspirator finish aspiration |
| Wipe | The surgical area is wiped with gauze for the purpose of wiping off fluid | Start: the gauze is grasped ready for wipe  End: finish a wipe on a target area |
| Clip | The tube structure is clamped by clip applier, including metal clip, absorbable clip and hem-o-lok | Start: the clip applier reaches the space  End: the clip applier is completely closed without clip fallen and leave the space |
| Cut | The tube structure is cut by scissor | Start: the scissor reaches the space  End: completely snip off the target tube |

# Supplementary Reference

1. Chen Z., An J., Wu S., Cheng K., You J., Liu J., et al. Surgesture: a novel instrument based on surgical actions for objective skill assessment. Surg Endosc. 2022;36(8):6113-6121.
