# Supplementary material for: Decoding surgical skill: an objective and efficient algorithm for surgical skill classification based on surgical gesture features –experimental studies
Source: Int J Surg. 2023 Dec 11;110(3):1441–9. doi: 10.1097/JS9.0000000000000975 (PMC10942222; doi:10.1097/JS9.0000000000000975)
Supplement: SUPPLEMENTARY MATERIAL [file js9-110-1441-s004.docx]

| **Table S2. The description of 63 Surgesture features.** |
| --- |
| **Counts (20) and duration (20) of Surgestures**  hook, hook dissection, blunt dissection, inefficient hook, scratch, coagulation, tamponade, aspiration, wipe, clip, cut  L-grasp, L-push, L-inefficient grasp, R-grasp, R-push, R-inefficient grasp  L-counts, R-counts, total-counts |
| **Interval duration of two Surgestures (12)**  L Max, Min, Mean, SD  R Max, Min, Mean, SD  Two hands Max, Min, Mean, SD |
| **Duration of MHT and DGB (1)**  Operation time |
| **Frequency of Surgestures shift during MHT and DGB (3)**  L Surgesture shift frequency  R Surgesture shift frequency  Two-hands Surgesture shift frequency |
| **D/E Classification (7)**  dissection Surgesture counts, exposure Surgesture counts, and their D/E ratio  dissection Surgesture duration, exposure Surgesture duration, and their D/E ratio  shift frequency of dissection and exposure |
