# Supplementary material for: Decoding surgical skill: an objective and efficient algorithm for surgical skill classification based on surgical gesture features –experimental studies
Source: Int J Surg. 2023 Dec 11;110(3):1441–9. doi: 10.1097/JS9.0000000000000975 (PMC10942222; doi:10.1097/JS9.0000000000000975)
Supplement: SUPPLEMENTARY MATERIAL [file js9-110-1441-s005.docx]

**Table S3.** **The comparison of checks for cystic stone among groups.**

| **Group** | **Total** | **check for cystic stone** | | **chi-square test** | |
| --- | --- | --- | --- | --- | --- |
|  |  | **No** | **Yes** | **chi-square** | **P.value** |
| Top | 16 | 12 (75.0%) | 4 (25.0%) | 7.951 | 0.019 |
| Medium | 33 | 12 (36.4%) | 21 (63.6%) |  |  |
| Bottom | 26 | 9 (34.6) | 17 65.4%) |  |  |
