# Supplementary material for: Decoding surgical skill: an objective and efficient algorithm for surgical skill classification based on surgical gesture features –experimental studies
Source: Int J Surg. 2023 Dec 11;110(3):1441–9. doi: 10.1097/JS9.0000000000000975 (PMC10942222; doi:10.1097/JS9.0000000000000975)
Supplement: SUPPLEMENTARY MATERIAL [file js9-110-1441-s007.docx]

**Table S4. The correlation among surgical skill assessment between surgeon-related factors and CVS scores.**

| **Group** | **DP** | **BD** | **Eff** | **TH** | **mGOALS** |
| --- | --- | --- | --- | --- | --- |
| **Hospital level** | -0.128 | -0.195 | -0.131 | 0.318 | 0.092 |
| **Work_years** | **0.373*** | 0.319 | 0.229 | 0.088 | 0.181 |
| **Case_experience** | 0.31 | 0.176 | 0.203 | 0.289 | 0.309 |
| **Title** | 0.155 | 0.21 | 0.116 | 0.071 | 0.117 |
| **Age** | 0.155 | 0.089 | -0.002 | -0.05 | -0.051 |
| **CVS** |  |  |  |  |  |
| **Total scores** | -0.025 | 0.146 | 0.199 | 0.123 | 0.163 |
| **Accomplishment** | 0.025 | -0.021 | -0.056 | 0.062 | 0.012 |

DP, depth perception; BD, bimanual dexterity; Eff, efficiency; TH, tissue hande.
